# Deep learning-enabled detection of hypoxic–ischemic encephalopathy after cardiac arrest in CT scans: a comparative study of 2D and 3D approaches

**DOI:** 10.3389/fnins.2024.1245791

**Published:** 2024-02-14

**Authors:** Noah S. Molinski, Martin Kenda, Christoph Leithner, Jens Nee, Christian Storm, Michael Scheel, Aymen Meddeb

**Affiliations:** ^1^Department for Neuroradiology, Charité – Universitätsmedizin Berlin, Freie Universität Berlin and Humboldt-Universität zu Berlin, Berlin, Germany; ^2^Department of Neurology with Experimental Neurology, Charité – Universitätsmedizin Berlin, Freie Universität Berlin and Humboldt-Universität zu Berlin, Berlin, Germany; ^3^Berlin Institute of Health at Charité – Universitätsmedizin Berlin, BIH Biomedical Innovation Academy, Berlin, Germany; ^4^Department of Nephrology and Medical Intensive Care, Charité – Universitätsmedizin Berlin, Freie Universität Berlin and Humboldt-Universität zu Berlin, Berlin, Germany

**Keywords:** artificial intelligence, cardiac arrest, CT, hypoxic-ischemic encephalopathy, classification, GradCAM

## Abstract

**Objective:**

To establish a deep learning model for the detection of hypoxic–ischemic encephalopathy (HIE) features on CT scans and to compare various networks to determine the best input data format.

**Methods:**

168 head CT scans of patients after cardiac arrest were retrospectively identified and classified into two categories: 88 (52.4%) with radiological evidence of severe HIE and 80 (47.6%) without signs of HIE. These images were randomly divided into a training and a test set, and five deep learning models based on based on Densely Connected Convolutional Networks (DenseNet121) were trained and validated using different image input formats (2D and 3D images).

**Results:**

All optimized stacked 2D and 3D networks could detect signs of HIE. The networks based on the data as 2D image data stacks provided the best results (*S100:* AUC: 94%, ACC: 79%, *S50:* AUC: 93%, ACC: 79%). We provide visual explainability data for the decision making of our AI model using Gradient-weighted Class Activation Mapping.

**Conclusion:**

Our proof-of-concept deep learning model can accurately identify signs of HIE on CT images. Comparing different 2D- and 3D-based approaches, most promising results were achieved by 2D image stack models. After further clinical validation, a deep learning model of HIE detection based on CT images could be implemented in clinical routine and thus aid clinicians in characterizing imaging data and predicting outcome.

## 1 Introduction

Globally, up to six million people suffer from sudden cardiac arrest each year, with less than 10% surviving (Zeppenfeld et al., 2022). Hypoxic ischemic encephalopathy (HIE) is a major cause of mortality and long-term disability among survivors in the acute phase (Geocadin et al., 2019). Accurate neuroprognostication is crucial, and current guidelines recommend combining several prognostic factors to predict poor outcomes (Deutsche Gesellschaft für Neurologie e.V, 2017). Besides neurological examination, laboratory tests and electrophysiology, computed tomography (CT) of the brain is vital in predicting of neurological outcome in patients with suspected HIE. Cerebral edema with decreased attenuation of gray matter in CT is a typical finding in patients with severe HIE, and a loss of boundary between gray and white matter has been shown to be associated with poor outcome (Kjos et al., 1983).

The early detection of HIE is especially important for choosing the right treatment protocol because the approach for patients with HIE differs significantly from that of patients without HIE. For patients with HIE, the primary goal of treatment is to minimize brain damage and promote neurological recovery. This may involve therapeutic hypothermia to reduce metabolic activity and protect the brain (Nolan et al., 2015). Additionally, supportive care, including maintaining adequate blood pressure, oxygenation, and fluid balance, is essential. In contrast, patients without HIE typically receive supportive care and monitoring, with a focus on preventing complications from the cardiac arrest itself (Nolan et al., 2021).

A reliable prognostic factor for poor outcome is the cerebral gray–white matter ratio (GWR) (Scheel et al., 2013; Lee et al., 2015; Na et al., 2018; Streitberger et al., 2019). Several studies determined that a GWR below 1.16–1.22 highly predicted poor neurological outcome (Kim et al., 2013; Scheel et al., 2013; Cristia et al., 2014; Lee et al., 2015). Various approaches exist to assess the GWR and predict poor outcome with high specificity and low-to-moderate sensitivity, depending on imaging timing (Na et al., 2018). Commonly, GWR is measured through manual placement of up to 16 regions of interest (ROIs) by a neuroradiologist (Metter et al., 2011). The manual placement of ROIs is however time consuming and prone to interrater variability (Kenda et al., 2022). More recently, Kenda et al. developed a simplified method with an automated placement of atlas ROIs (bilateral putamen and internal capsule) with comparable outcome prediction to the 16 ROIs method (Kenda et al., 2021).

The recent development of machine and deep learning has significantly progressed and advanced the field of medical image analysis. Deep convolutional neural networks (CNNs) became widespread in the last decade and successfully addressed tasks such as object detection, image segmentation and classification. Several studies have demonstrated promising results in organ segmentation (Akkus et al., 2017; Livne et al., 2019; Hssayeni et al., 2020; Meddeb et al., 2021) and disease classification (Artzi et al., 2019; Burduja et al., 2020; Li et al., 2020; Meddeb et al., 2022; Nishio et al., 2022). In neuro-imaging, deep learning models have been successfully applied to intracranial hemorrhage detection and segmentation using CT images (Xu et al., 2021), as well as brain tumor classification using magnetic resonance imaging (MRI) (Gao et al., 2022).

The purpose of this investigation was to develop a deep learning framework capable of identifying imaging features of hypoxic–ischemic encephalopathy (HIE) in CT scans of resuscitated cardiac arrest patients. The principal emphasis was on investigating the feasibility and constraints of deep learning in HIE detection, along with technical and clinical prerequisites. Various model architectures were evaluated using 2D and 3D data formats to develop a state-of-the-art model with the highest achievable classification accuracy.

## 2 Methods

### 2.1 Study design

This retrospective observational study used prospectively collected data from adult (aged ≥18 years) comatose CA survivors treated with targeted temperature management (TTM) at a single tertiary academic hospital between 2010 and 2019. This study was approved by the institutional review board of the Charité (No.: EA2/066/17, EA4/136/21). The recommendations of the CLAIM checklist of the RSNA and the DECIDE-AI checklist were largely adhered to and are attached as Supplementary materials (Mongan et al., 2020; Vasey et al., 2022). Due to the retrospective design of this study, new informed consent was not required. All patient data was strictly protected and anonymized prior to analysis.

### 2.2 Study population

We included 168 patients from a previously published cohort of 483 cardiac arrest (CA) survivors with suspected HIE from our institution (Kenda et al., 2021). After admission to the intensive care unit (ICU), patients were treated with TTM (body temperature of 33°C for 24 h) according to the European Resuscitation Council Guidelines (Nolan et al., 2015). All patients received CT-imaging within seven days after *CA.* The head CT images were taken by several GE Lightspeed and Revolution scanners as well as on Toshiba Aquilion. Neurological outcome was assessed by treating physicians at hospital discharge using the cerebral performance category (CPC) scale. For prognostic evaluation in this study, the outcome was dichotomized into “good” (CPC 1–3) and “poor” outcome (CPC 4–5). A board-certified radiologist (AM) and a board-certified neuroradiologist (MS) classified CTs with the labels “HIE” or “no HIE.” Both radiologists were blinded to the clinical parameters of the study population. All patient data was handled only using anonymized identifiers based on patient cohort and a number in the form of X123 which still enables future de-identification A graphical representation of the patient selection and data flow of this study can be found in the Supplementary Figure S1.

### 2.3 CT imaging characteristics and preprocessing

**Figure 1:**
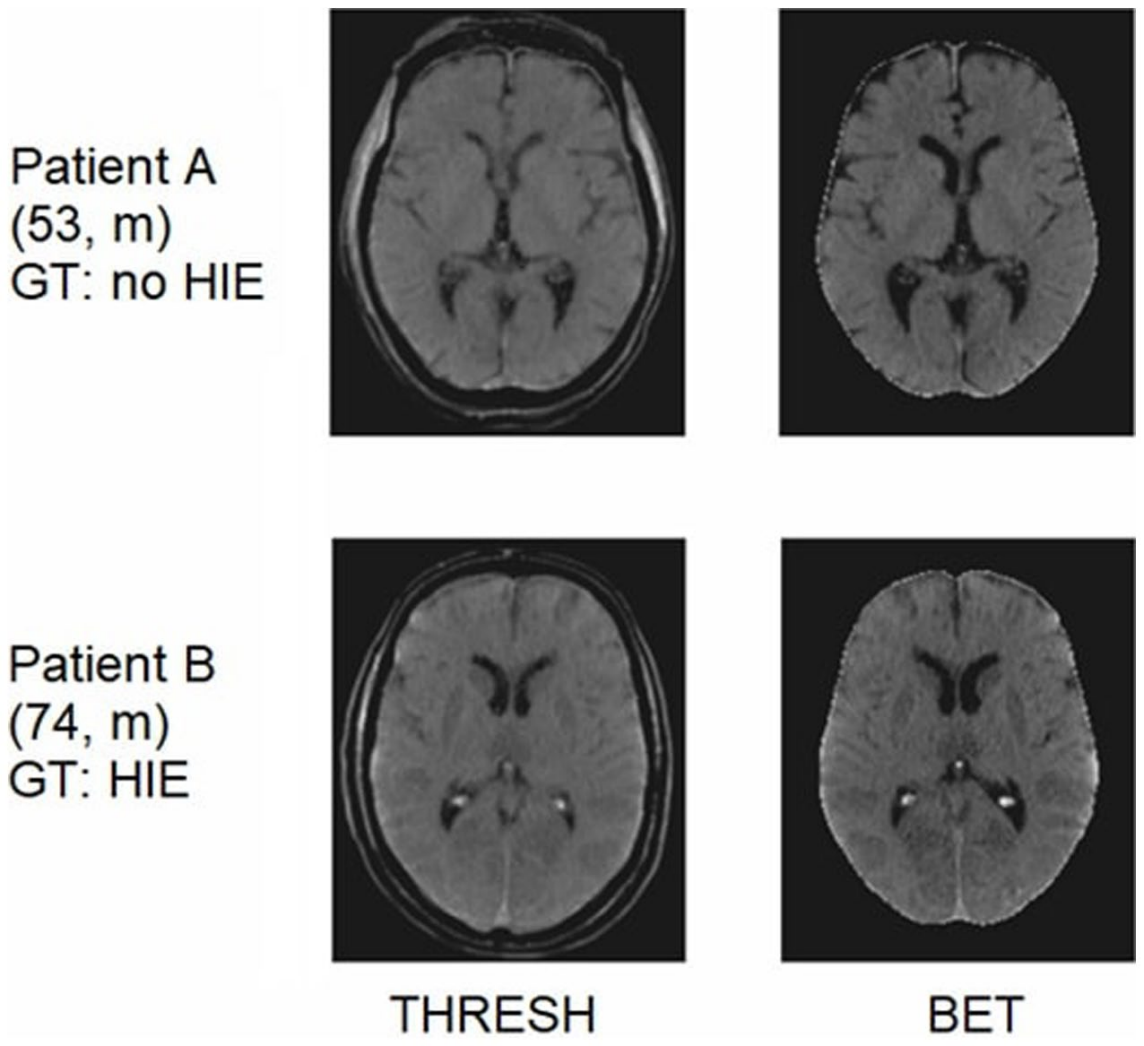
CT images of two different patients after passing through the two preprocessing pipelines. Patient A showing no signs of HIE. Patient B showing signs of severe HIE (“reversal sign”). GT, ground truth.

All CT images were reformatted from standard DICOM to Neuroimaging Informatics Technology Initiative (NIfTI) format. In a second stage, they were co-registered in a linear and non-linear mode to a standardized CT template in an MRI-based standard space using FNIRT and FLIRT functions from FSL (FMRIB Software Library v6.0, FMRIB, Oxford, UK) (Jenkinson et al., 2012). To reduce superfluous information, we evaluated different preprocessing techniques: The first technique consisted of thresholding the skull bone and obtaining CT images with brain and head ridge visible (THRESH), the second technique used the FSL brain extraction tool (BET). Following standard best practices of the train-test split for machine learning models, 134 CTs were used for training and validation, and the remaining 34 CTs for independent testing after training. Examples of CT images obtained from both preprocessing pipelines with different degrees of HIE and no HIE are shown in Figure 1.

### 2.4 Deep learning models and input data

The 2D and 3D DenseNets presented in this paper were implemented using the Python programming language (version 3.7, Python Software Foundation^1^) on the open-source deep learning framework MONAI (version 0.9^2^) in conjunction with PyTorch (version 1.13.0^3^).

**Figure 2:**
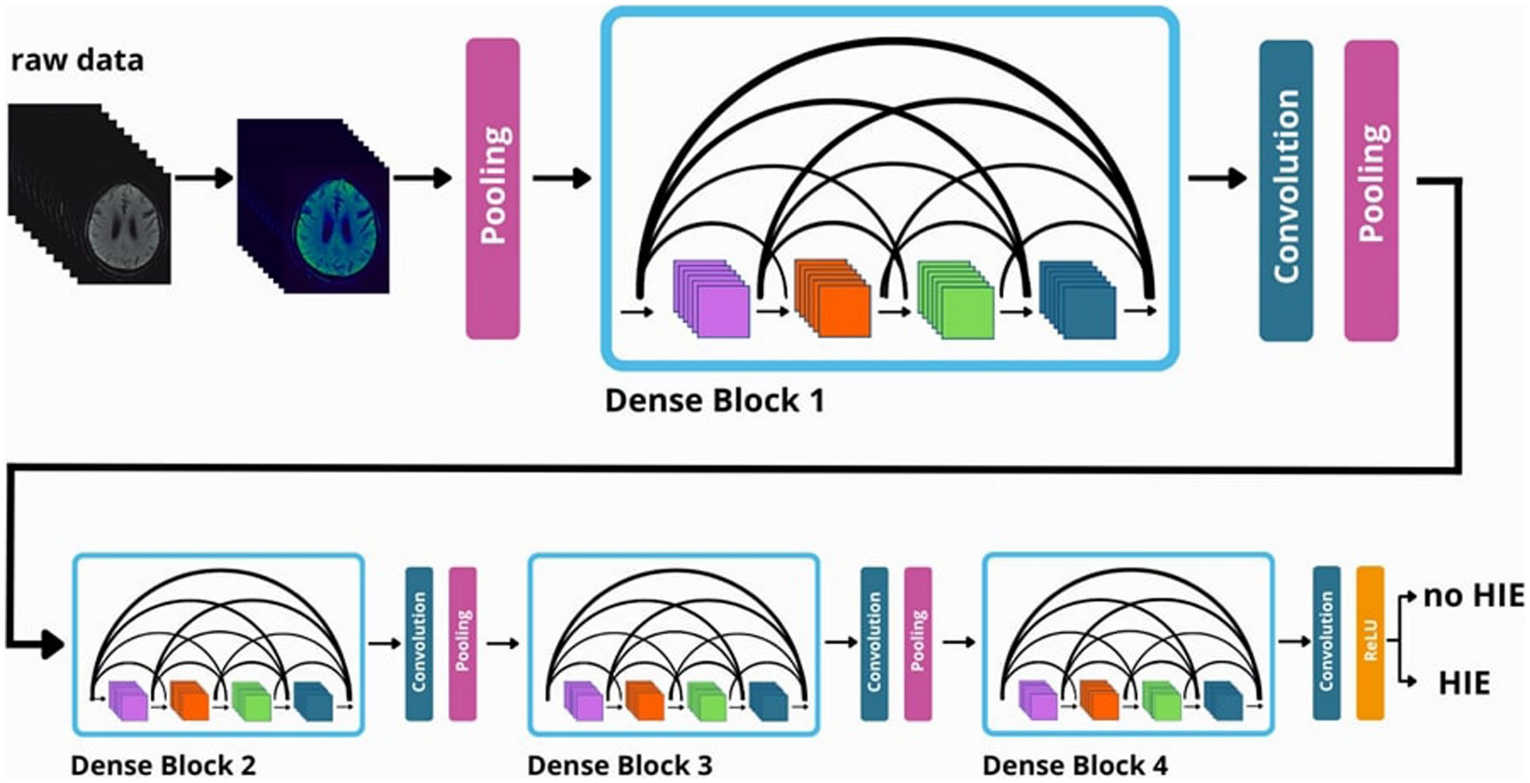
Visualization of the data processing flow in a 3D-DenseNet network.

All these networks are based on DenseNet121 architecture, which is characterized by four dense blocks with three transition layers and a final classification layer (Huang et al., 2016). DenseNets show a very high performance in deep learning classification (Zhou et al., 2022). Figure 2 shows a schematic diagram of the data processing within the neural network.

To determine the highest classification performance, five networks were trained with different data formats for both the BET and THRESH preprocessed data:

- 3D-NET-ALL: all CT data as 3D images
- 2D-NET-ALL: all CT data as 2D images
- 2D-NET-*S100*: a stack of 100 2D images per CT scan (from the skull base until centrum semiovale)
- 2D-NET-*S50*: a stack of 50 2D images per CT scan (50 slices containing basal ganglia)
- 2D-NET-BG: one slice at the level of the anterior commissure

**Figure 3:**
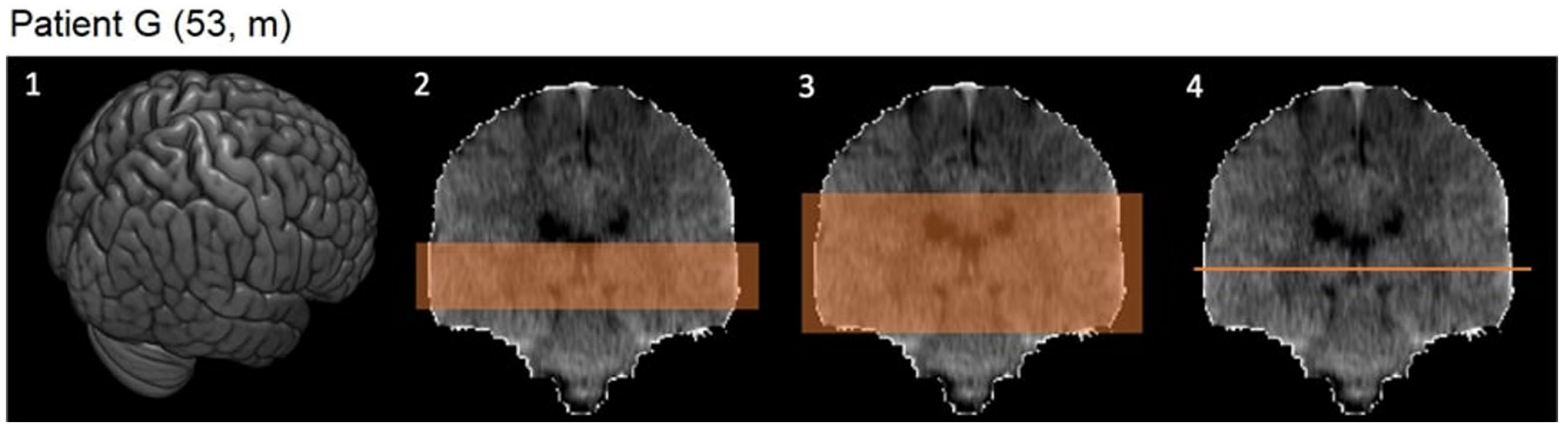
CT images in 3D and coronal reconstruction. 1: 3D image of the whole brain. 2: Image stack of 50 slices including the basal ganglia. 3: Image stack of 100 slices. 4: One slice at the level of the anterior commissure.

Evidence from previous studies (Singh et al., 2020) indicates that certain layers of the CT images contain significantly more relevant information than others, which is why a 2D model with all images (2D-NET-all, 181 images per CT), two 2D models with image stacks (2D-NET-*S100*, 100 images per CT; 2D-NET-*S50*, 50 images per CT) and one model with only a single slice at the level of the anterior commissure (2D-NET-BG, 1 slice per CT) were tested. A schematic visualization of the used images per CT scan can be found in Figure 3.

The preprocessed data were subjected to various transformations such as intensity scaling, rescaling, and rotations to obtain a higher variance of the input data. During training, a cross entropy loss function was used (Selvaraju et al., 2016). As a hyperparameter of interest, the learning rate was fine-tuned for each model in the range of 10^−5^ to 10^−2^. This was achieved by using the Adam optimizer and determining the steepest gradient of the loss function over the learning rate. Each model was trained for a maximum of up to 100 epochs. For internal validation after each epoch, a subset of 34 CT scans was used. The best training epoch was determined based on the highest AUC value. To optimize for highest specificity/lowest FPR, the threshold for the decision certainty of the model was increased from 0.5 to 0.95 resp. 0.99. Subsequently, the result was obtained by binarizing the probability values using the adjusted threshold. Additionally, GradCAM images were created for visual verification of the model predictions (Selvaraju et al., 2016).

### 2.5 Statistical analysis

Continuous variables between two groups were compared using a Welch’s t-tests or Mann–Whitney U tests according to the normality of the data. Classification performances were evaluated using the area under the receiver operating characteristic curve (AUC), accuracy (ACC), sensitivity (SEN), and specificity (SPE) for the training and test set. Inter-rater reliability was determined using Cohen’s kappa (k) for nominal variables, such as the presence/absence of HIE signs on CT images and classified according to Altmann’s scheme (Altman, 1990). *p-*values <0.05 were considered statistically significant at 95% CIs. Statistical analysis was performed using Python 3.7, the scipy library [version 1.8.1^4^ (Pedregosa et al., 2012) and R Studio (Version 2022.12.0+353)].

## 3 Results

### 3.1 Study population

Of 168 patients, 50 (29.8%) were female, the average age was 60 (±12) years. According to the labeling of the neuroradiologist, 88 (52.4%) showed signs of HIE in their CT images while the other 80 (47.6%) showed no signs of HIE. Among all patients, 128 (76.2%) of CT images were from patients with out-of-hospital cardiac arrests (OHCA). While almost 87 (98.9%) patients labeled with “HIE” by the radiologist had poor outcome (CPC 4–5 at hospital discharge), the “no HIE” group included 22 (25.0%) cases with CPC 4–5. An overview of the analyzed demographic and clinical characteristics of the study population is shown in Table 1.

**Table 1:** Demographic and clinical characteristics of the study population.

Parameter	Total	HIE	no HIE	*p*-value*
*n*	168	88	80	
Age	60 [48–70]	57 [44–68]	63 [54–72]	0.021
Sex				
Male	118 (70.2%)	60 (68.2%)	58 (72.5%)	0.271
OHCA	128 (76.2%)	73 (83.0%)	55 (68.8%)	0.033
Shockable Rhythm (*n* = 167/88/79)	78 (46.7%)	37 (42.0%)	41 (51.9%)	0.205
Primary cause of arrest (*n* = 166/87/79)				0.075
Cardiac	69 (41.6%)	29 (33.3%)	40 (50.6%)	
Respiratory	39 (23.5%)	24 (27.6%)	15 (19.0%)	
Other **	58 (34.9%)	34 (39.1%)	24 (30.4%)	
Time to ROSC (min) (*n* = 157/81/76)	20 [10–30]	22 [15–60]	12 [8–21]	0.007
Total Adrenalin Dose (mg) (*n* = 157/87/70)	2 [1–5]	4 [2–7]	2 [1–3]	<0.001
APACHE Score (*n* = 165/86/79)	36 [30–40]	37 [31–41]	34 [29–39]	0.1
Length of ICU stay (days)	10 [5–17]	5 [3–11]	15 [10–28]	<0.001
Time on Ventilator (hours)	170 [96–334]	123 [60–235]	242 [135–432]	0.003
CT acquisition (hours after CA) (*n* = 167/88/79)	20 [3–93]	18 [3–85]	34 [4–125]	0.004
Neurological outcome at hospital discharge				<0.001
CPC 1	36	0	36	
CPC 2	22	0	22	
CPC 3	4	1	3	
CPC 4	11	9	2	
CPC 5	95	78	17	

*Mann–Whitney U test (resp. Welch’s *t*-tests). **Intoxication, metabolic and unknown cases.

To verify the correct classification between the two groups, 20% of the CT images were reviewed by a second board-certified neuroradiologist and the interobserver variability was determined. The independent radiological reviews yielded a Cohens kappa of 0.758, which is a “good agreement” according to Altmann’s scheme.

For missing data, the parameters are calculated on the basis of the available data and their new total number is specified.

### 3.2 Evaluation of classification performance and further metrics

Overall, the 2D-NET-*S100* and 2D-NET-*S50* achieved the highest AUCs (AUC: 94% resp. 93%) and accuracies (both ACC: 79%), 2D-NET-all (AUC: 89%, ACC: 76%) performed worse than the stack models but still a lot better than the 3D-NET-all (AUC: 70%, ACC: 50%). The 2D-NET-BG (AUC: 47%, ACC: 50%) performed on the same level as a random guess. The receiver operating characteristic curves with their corresponding AUCs are shown in Supplementary Figure S2. An overview of the most relevant performance metrics for all networks on the BET data can be found in Table 2. Networks using the THRESH data performed less stable than on the BET data. A table of all performance parameters for all networks including both kinds of preprocessed input data as well as two probability thresholds is displayed in the Supplementary Table S1.

**Table 2:** Overview of the various key metrics for comparing the different neuronal networks on the BET data.

Parameter	3D-NET-all	2D-NET-all	2D-NET-*S100*	2D-NET-*S50*	2D-NET-BG
SEN [%]	0	53	59	59	0
SPE [%]	100	100	100	100	100
ACC [%]	50	76	79	79	50
AUC [%]	70	89	94	93	47

All metrics are based on the test dataset including 34 CT scans and a network probability threshold of 0.99. SEN, sensitivity; SPE, specificity; ACC, accuracy; AUC, area under the curve.

### 3.3 Deep learning visualization

**Figure 4:**
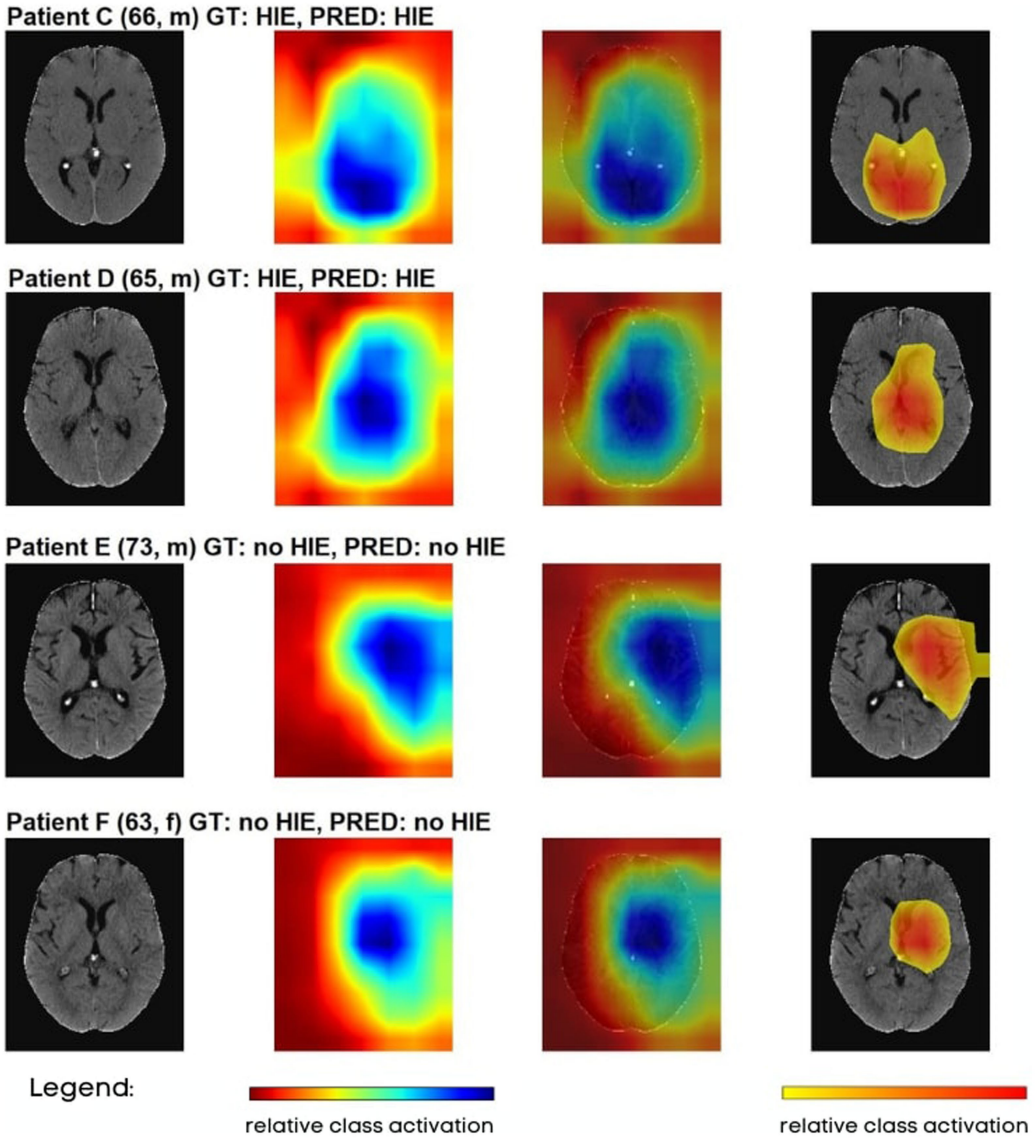
Visualization of various CT images tested on 2D-NET-*S100* network, where the test images were partially overlaid with GradCAM images. GT, ground truth; PRED, prediction.

To better understand the decision-making process of our models, Gradient-weighted Class Activation Mapping (GradCAM) was implemented and evaluated for each patient. GradCAM utilizes the activation gradient information of the last convolutional layer to highlight the regions of highest importance for the prediction of the model and visualizes these regions similar to heatmaps (Selvaraju et al., 2016). Figure 4 contains examples of GradCAM images implemented on 2D THRESH Ct scans. The first images on the left-hand side depict the original CT scans. The second image in each row presents a comprehensive GradCAM image. The third image overlays the entire GradCAM image with 60% opacity onto the original CT scan. The fourth image showcases the region encompassing the 30th percentile of the most vigorous activations in the Gradcam images, superimposed over the original CT. All test data is visualized in the same manner in Supplementary Figure S3.

In general, the GradCAM visualizations showed that the highest information density for the decision on the presence of HIE was based on the expression strands of the sulci in the area of the centrum semiovale and the basal ganglia. This presentation was quite analogous for all true-positive and true-negative cases. With the exception of true-positive cases, in which no sulci were visible and the GWR was already visually expected to be very low, further brain areas were also displayed.

Furthermore, the GradCAM images also made it possible to determine that false positive or false negative cases mostly marked areas outside the brain or the limits of the FSL brain extraction tool.

## 4 Discussion

The aim of this study was to develop a convolutional neural network (CNN) that could detect HIE in CA patients and to explore which data types and network architectures are best to establish a state-of-the-art model. All trained models were able to detect HIE with varying accuracies. Both 2D-NETs (*S100*, *S50*) trained on image stacks from brain extracted images (BET-images) achieved best predictive performance and highest accuracy in the training/validation as well as in the test set.

In recent years, CT imaging has played a central role in a multimodal approach to estimate the prognosis and consequently decide on continuation or withdrawal of life-sustaining therapy in CA patients. Grey-White Matter Ratio (GWR) measurement has proven to be a strong prognosticator of poor outcome with high specificity and low- to moderate sensitivity (Lee et al., 2015; Na et al., 2018). However, GWR measurement needs neuroradiological expertise that is rarely found outside of referral care centers and university hospitals (Elmer et al., 2023). In this study, we established a deep learning model for detection of HIE signs on CT images that can be easily implemented in clinical routine.

We tested two different preprocessing pipelines for our data. The first pipeline involved thresholding to strip the skull, while the second pipeline involved brain extraction. The use of thresholding technique in the first pipeline was faster to implement but resulted in some prediction bias. This may have been due to the presence of superfluous information through the head ridge, e.g., subcutaneous edema that could indicate continuous heart failure after CA and lead to a poor outcome regardless of HIE. On the other hand, brain extraction removed superfluous information, but resulted in some partial information loss through removal of cerebrospinal fluid, veins, and superficial cortex. The brain extraction technique was found to produce better predictive performance as well as a more plausible predictions judging from the GradCAM images.

After performing the necessary preprocessing steps, we trained multiple models using different data formats, including 3D images, 2D images of the entire brain, 2D stacks of the most significant brain regions, and 2D slices at the level of the anterior commissure. Our results demonstrated that networks trained on the 2D image stacks, which included 50 resp. 100 image slices but not all images of each CT scan, delivered the best performance. One possible explanation for this is the significantly larger amount of training data available for the 2D stack format, with 13,400 images (for 2D Stack-100) compared to only 134 images for the 3D data in the training set. Our results are corroborated by Crespi et al., who described that 2D networks outperformed 3D networks on medical data despite the lower number of parameters (Crespi et al., 2022). These findings highlight the importance of carefully selecting the appropriate data format for training CNN models in medical imaging applications to achieve optimal performance.

Moreover, GradCAM visualization provides a valuable tool for understanding the decision-making process of the CNN models. These maps helped us gain insight into the reasoning behind the model’s predictions and identify areas that may require further investigation or attention from treating clinicians. According to our GradCAM images, our models predominantly use information in the basal ganglia and cortical sulci when discriminating between HIE and no HIE. To further analyze this observation systematically, we divided the GradCAMs into subgroups based on the model’s decisions (true positive, true negative, false negative, false positive) and visualized them again as group overlay plots. These visualizations are presented in Supplementary Figure S4, where the metrics of the subgroups correspond to rows 34–37 of columns I and J in Supplementary Table S1. First, the overlays of the THRESH method highlight significantly more areas from outside the brain than in the brain extraction group (BET), which was the main reason why we developed the BET pipeline. The subgroup overlays indicate that for both types of CT data, similar regions were highlighted for both true positive and false positive decisions, as well as for both true negative and false negative decisions, respectively. While the heatmaps for true positive focuses more on deep grey matter including basal ganglia, the heatmaps in true negative are more diffused, suggesting the model is not focusing on any particular area indicative of HIE. This observation emphasizes again the critical importance of interpretability neural networks for medical imaging analysis, as it can help identify false predictions caused by information loss or other factors.

In addition, we also complied with 37 of the 42 checklist items on the RSNA’s CLAIM checklist during the study. The open items were either not applicable and indicated external validation, which we have already identified as a limitation of our study and will be addressed in future work. Of the DECIDE-AI checklist, 28 of the 37 items were adhered to. The open points are all in the area of implementation in everyday clinical practice and usability by other medical users, which was not yet planned as part of this study. We would like to point out that the scope of this checklist is aimed at other areas than this initial proof of concept study was intended to investigate.

In a recent publication by Mansour et al. (2022), machine and deep learning were utilized to identify patients who would exhibit radiologic evidence of apparent HIE on follow-up CT scans. Although this study demonstrates the potential of deep learning in detecting features that may not be visible to human raters, their proposed method included various significant limitations (i.e., high risk of overfitting due to small data set, questionable training pipeline and principal component analysis), which could result in partially erroneous results (Molinski et al., 2022). Our approach involved training our deep learning models from scratch, with direct class prediction as the output, without manual feature selection or additional machine learning modeling. Additionally, we believe that interpretability of the model’s predictions is crucial, which is why we utilized GradCAM visualization.

Our deep learning-based classification method differs significantly from GWR measurement. Unlike GWR, which relies on placing ROIs in the basal ganglia and white matter and may miss important information in other regions such as the cerebellum, our model considers all spatial information in the images. In addition, GWR only takes into account the Hounsfield units of the ROI, neglecting other relevant anatomical factors such as sulcal relief and ventricular enlargement. Our multi-class deep learning classification method uses a neural network with output nodes equal to the number of classes (in our case two: “HIE” and “no HIE”). Each output node is associated with a class and generates a score for that class, which is then passed through an activation layer to obtain probability values. As prediction probability threshold is set at 0.5 by default, we adjusted for the optimal threshold to achieve 0% FPR, which is necessary for clinical implementation (Geocadin et al., 2019).

Our study has several potential limitations that should be considered. First, as the study has a retrospective, single-center design, our model has yet to be externally and prospectively validated. Furthermore, our cohort of 168 patients is relatively small, and a larger dataset is needed to ensure the reproducibility of results and reduce the risk of overfitting. We tried to reduce this risk as much as possible by utilizing raw image transforms, a learning rate finder and an Adam gradient optimizer. Additionally, choosing a standard training-validation split instead of cross-validation can also be seen as limitation, as cross-validation could probably deliver a more robust assessment of generalization ability and facilitate a more comprehensive evaluation of hyperparameters. But we still opted for a conventional training-test split instead of employing cross-validation in this proof-of-concept study, because (i) this study was designed to minimize computational overhead, (ii) repeatedly training and evaluating the model on different subsets of a small dataset may cause the model to memorize specific patterns rather than learning the underlying patterns of the data and thereby increases the risk of overfitting and (iii) a standard training-test split allows for a more straightforward visualization of the model’s performance and thus enables better comparability of the different models used in the study. Another point of concern is the choice of ground truth: In our study, we used neuroradiological expertise as the ground truth for “HIE” and “no HIE” labels. However, this may not necessarily reflect the underlying pathology or clinical status of the patients. The main reason for this choice is the difficulty to clinically determine the real cause of poor outcome, as many patients with HIE develop other complications such as cardiac or pulmonary complications, which may lead to death. Given the complexity of clinical cases like HIE, it is important to note that the ground truth of our training data corresponds to the expert opinion of a radiologist, which may already contain errors. To minimize this risk, a portion of the data was reviewed by another neuroradiologist to determine interrater variability. Our analysis found a high level of agreement between raters, which was significantly better than what is typically observed for HIE (Caraganis et al., 2020). However, there is still a small possibility that the ground truth of certain images may be incorrect. Another limitation is the quality of the data itself. Despite various preprocessing techniques, there is always the possibility that the individual CTs are not sufficiently homogenized since our data originated from three different scanner types of two manufactures. In a phantom study, Li et al. (2021) showed a high variability of image quality between different CT scanners. Roa et al. (2015) demonstrated that the image quality of a same CT scanner decreased over time. The data quality is also partially impaired by variability in patient characteristics, such as age and the timing of the CT scan after *CA.* It is well known that age-related cerebral atrophy and hypoattenuation of white matter in chronic small vessel ischemic disease can complicate neuroradiological diagnosis. Furthermore, HIE diagnosis on later CT scans is also significantly more sensitive for poor outcomes (GWR decreases over time in patients with severe HIE), so the sensitivity for prediction of poor outcome is higher for late CTs (>24 h after CA) as compared to early CTs (<6 h after CA) (Streitberger et al., 2019). As these aspects confound the outcome prognosis for a human rater, they also confound the training of a neural network, especially on a small dataset.

For our future work, we first want to address the current limitations especially in regards to the data sampling (i.e., cross-validation) and retest and retrain it on a larger dataset. We will also explore newer techniques of CT quality harmonization such as the ComBat method (Johnson et al., 2007; Orlhac et al., 2021). Moreover, as the timing of brain computed tomography and accuracy of outcome prediction are correlated, we will investigate the influence of CT timing on the predictive performance of our model. Our vision is to develop a multimodal model, for which we will integrate further parameters such as serum biomarkers and electrophysiology data to improve outcome prediction. Transfer learning in combination with an external multi-center validation approach can be used to further optimize this pilot study (Weiss et al., 2016).

## 5 Conclusion

In this study we established a state-of-the-art, deep learning-based model for detection of hypoxic–ischemic encephalopathy on CT images which can be trained on 2D or 3D images.

The best performance was achieved by neuronal networks trained on 2D image stacks of brain extracted CT data. After implementing the described improvements and external validation, our model can be implemented in clinical routine and help clinicians with outcome prediction of HIE in CA patients.

## Data availability statement

The data analyzed in this study is subject to the following licenses/restrictions: the developed code, additional data and/or materials will be disclosed upon reasonable request to the corresponding author. Requests to access these datasets should be directed to noah.molinski@charite.de.

## Ethics statement

The studies involving humans were approved by Institutional review board of the Charité (Nos.: EA2/066/17, EA4/136/21). The studies were conducted in accordance with the local legislation and institutional requirements. Written informed consent for participation was not required from the participants or the participants’ legal guardians/next of kin in accordance with the national legislation and institutional requirements.

## Author contributions

NM: conceptualization, formal analysis, investigation, methodology, software, visualization, writing – original draft, and writing – review and editing. MK: data curation, formal analysis, methodology, software, and writing – review and editing. CL: data curation, methodology, and writing – review and editing. JN: data curation and writing – review and editing. CS: data curation and writing – review and editing. MS: conceptualization, formal analysis, project administration, supervision, and writing – review and editing. AM: conceptualization, formal analysis, project administration, software, visualization, writing – original draft, and writing – review and editing. All authors contributed to the article and approved the submitted version.

## Footnotes

^1^https://www.python.org (accessed on 18 July, 2022).

^2^ https://docs.monai.io

^3^ https://pytorch.org

^4^ https://scipy.org/
