# Supplementary material for: Deep learning-enabled detection of hypoxic–ischemic encephalopathy after cardiac arrest in CT scans: a comparative study of 2D and 3D approaches
Source: Front Neurosci. 2024 Feb 14;18:1245791. doi: 10.3389/fnins.2024.1245791 (PMC10899383; doi:10.3389/fnins.2024.1245791)
Supplement: Supplementary file 1 [file Data_Sheet_1.PDF]

CLAIM: Checklist for Artificial Intelligence in Medical Imaging

Project: Deep Learning-Enabled Outcome Prediction of Hypoxic-Ischemic Encephalopathy in CT scans: A Comparative Study of 2D and 3D Approaches

Section / Topic	No.	Item	
**TITLE / ABSTRACT**			
	**1**	Identification as a study of AI methodology, specifying the category of technology used (e.g., deep learning)	**yes**
	**2**	Structured summary of study design, methods, results, and conclusions	**yes**
**INTRODUCTION**			
	**3**	Scientific and clinical background, including the intended use and clinical role of the AI approach	**yes**
	**4**	Study objectives and hypotheses	**yes**
**METHODS**			
Study Design	**5**	Prospective or **retrospective** study	**yes**
	**6**	Study goal, such as model creation, exploratory study, feasibility study, non-inferiority trial	**yes**
Data	**7**	Data sources	**yes**
	**8**	Eligibility criteria: how, where, and when potentially eligible participants or studies were identified (e.g., symptoms, results from previous tests, inclusion in registry, patient-care setting, location, dates)	**yes**
	**9**	Data pre-processing steps	**yes**
	**10**	Selection of data subsets, if applicable	**yes**
	**11**	Definitions of data elements, with references to Common Data Elements	**yes**
	**12**	De-identification methods	**yes**
	**13**	How missing data were handled	**yes**
Ground Truth	**14**	Definition of ground truth reference standard, in sufficient detail to allow replication	**yes**
	**15**	Rationale for choosing the reference standard (if alternatives exist)	**yes**
	**16**	Source of ground-truth annotations; qualifications and preparation of annotators	**yes**
	**17**	Annotation tools	**---**
	**18**	Measurement of inter- and intrarater variability; methods to mitigate variability and/or resolve discrepancies	**yes**
Data Partitions	**19**	Intended sample size and how it was determined	**yes**
	**20**	How data were assigned to partitions; specify proportions	**yes**

	**21**	Level at which partitions are disjoint (e.g., image, study, patient, institution)	**yes**
Model	**22**	Detailed description of model, including inputs, outputs, all intermediate layers and connections	**yes**
	**23**	Software libraries, frameworks, and packages	**yes**
	**24**	Initialization of model parameters (e.g., randomization, transfer learning)	**yes**
Training	**25**	Details of training approach, including data augmentation, hyperparameters, number of models trained	**yes**
	**26**	Method of selecting the final model	**yes**
	**27**	Ensembling techniques, if applicable	**----**
Evaluation	**28**	Metrics of model performance	**yes**
	**29**	Statistical measures of significance and uncertainty (e.g., confidence intervals)	**yes**
	**30**	Robustness or sensitivity analysis	**yes**
	**31**	Methods for explainability or interpretability (e.g., saliency maps), and how they were validated	**yes**
	**32**	Validation or testing on external data	**---**
**RESULTS**			
Data	**33**	Flow of participants or cases, using a diagram to indicate inclusion and exclusion	**yes**
	**34**	Demographic and clinical characteristics of cases in each partition	**yes**
Model performance	**35**	Performance metrics for optimal model(s) on all data partitions	**yes**
	**36**	Estimates of diagnostic accuracy and their precision (such as 95% confidence intervals)	**yes**
	**37**	Failure analysis of incorrectly classified cases	**yes**
**DISCUSSION**			
	**38**	Study limitations, including potential bias, statistical uncertainty, and generalizability	**yes**
	**39**	Implications for practice, including the intended use and/or clinical role	**yes**
**OTHER INFORMATION**			
	**40**	Registration number and name of registry	**----**
	**41**	Where the full study protocol can be accessed	**----**
	**42**	Sources of funding and other support; role of funders	**yes**

Based on: Mongan J, Moy L, Kahn CE Jr. Checklist for Artificial Intelligence in Medical Imaging (CLAIM): a guide for authors and reviewers. Radiol Artif Intell 2020; 2(2):e200029. https://doi.org/10.1148/ryai.2020200029
