# Supplementary figures and images for: Deep learning-enabled detection of hypoxic–ischemic encephalopathy after cardiac arrest in CT scans: a comparative study of 2D and 3D approaches
Source: Front Neurosci. 2024 Feb 14;18:1245791. doi: 10.3389/fnins.2024.1245791 (PMC10899383; doi:10.3389/fnins.2024.1245791)

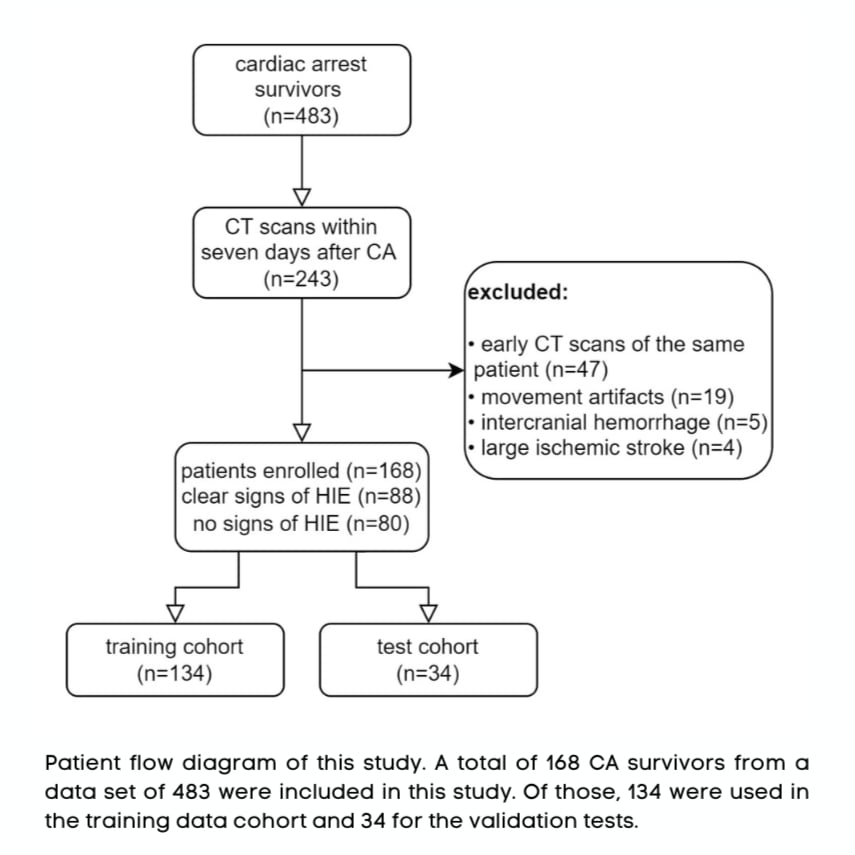

Supplement: Supplementary file 3 [file Image_1.JPEG]

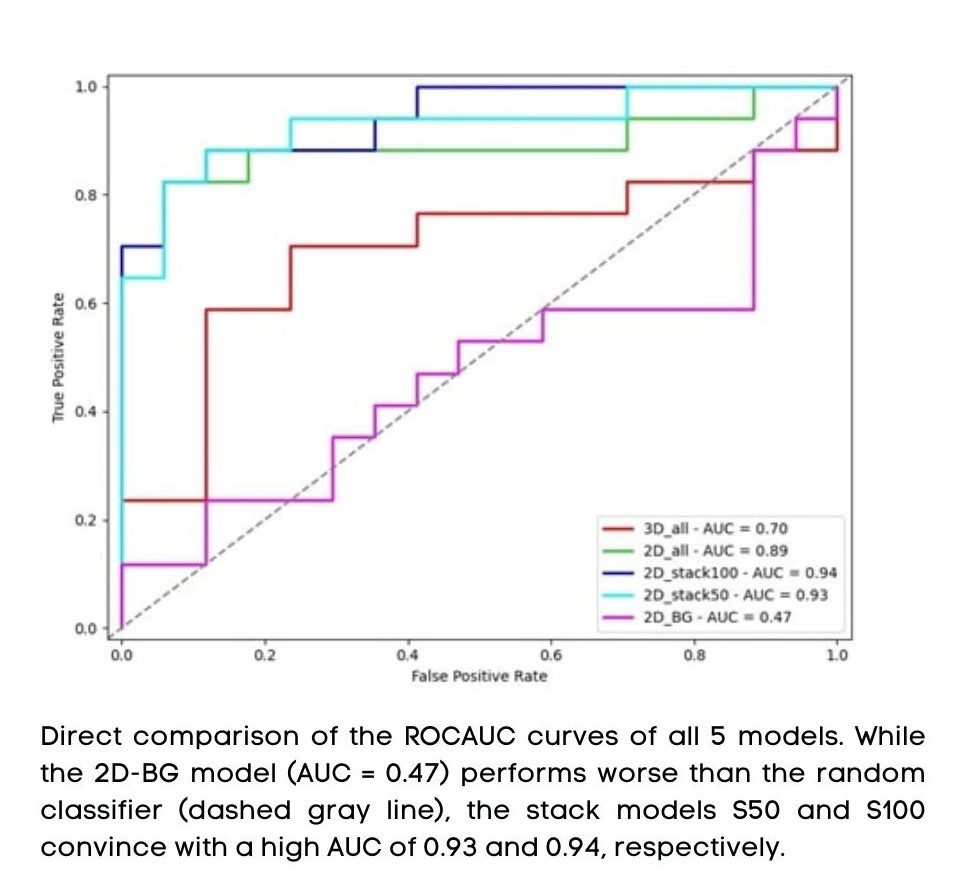

Supplement: Supplementary file 4 [file Image_2.JPEG]

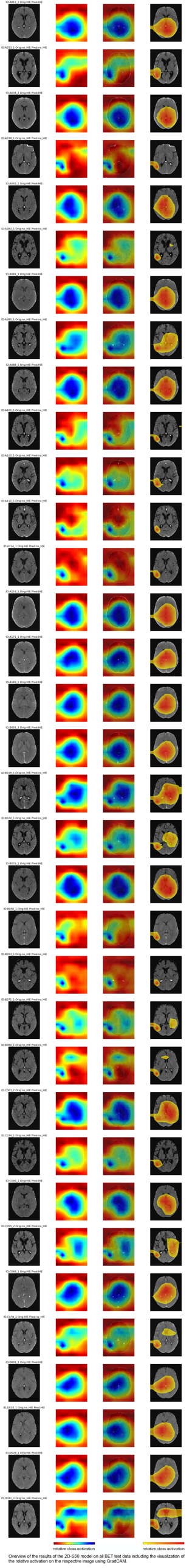

Supplement: Supplementary file 5 [file Image_3.JPEG]

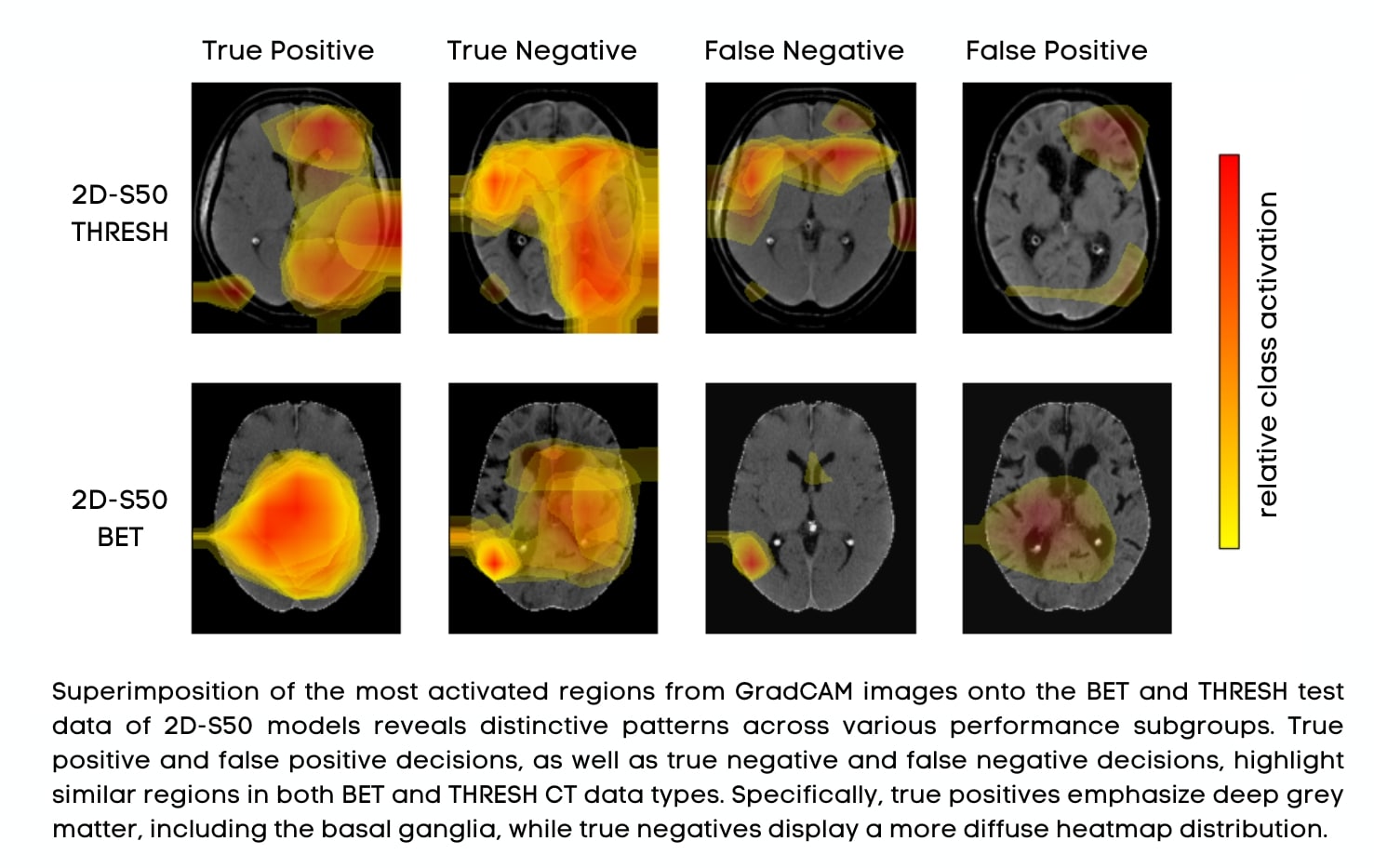

Supplement: Supplementary file 6 [file Image_4.JPEG]
